# Proposta de metodologia para estimar a área de cobertura potencial por equipes de atenção primária

**DOI:** 10.26633/RPSP.2019.47

**Published:** 2019-05-24

**Authors:** Thiago Augusto Hernandes Rocha, Dante Grapiuna de Almeida, Pedro Vasconcelos Maia do Amaral, Núbia Cristina da Silva, Erika Bárbara Abreu Fonseca Thomaz, Rejane Christine de Sousa Queiroz, Allan Claudius Queiroz Barbosa, João Ricardo Nickenig Vissoci

**Affiliations:** 1 Organização Pan-Americana da Saúde (OPAS) Organização Pan-Americana da Saúde (OPAS) Brasília(DF) Brasil Organização Pan-Americana da Saúde (OPAS), Brasília (DF), Brasil.; 2 Medomai Informática Medomai Informática Belo Horizonte(MG) Brasil Medomai Informática, Belo Horizonte (MG), Brasil.; 3 Universidade Federal de Minas Gerais (UFMG), Faculdade de Ciências Econômicas Universidade Federal de Minas Gerais (UFMG), Faculdade de Ciências Econômicas Departamento de Economia Belo Horizonte(MG) Brasil Universidade Federal de Minas Gerais (UFMG), Faculdade de Ciências Econômicas, Departamento de Economia, Belo Horizonte (MG), Brasil.; 4 Methods, Analytics and Technology for Health Consortium (MATH) Methods, Analytics and Technology for Health Consortium (MATH) Duke University Durham(NC) Estados Unidos Methods, Analytics and Technology for Health Consortium (MATH), Duke University, Durham (NC), Estados Unidos.; 5 Universidade Federal do Maranhão (UFMA) Universidade Federal do Maranhão (UFMA) Departamento de Saúde Coletiva São Luís(MA) Brasil Universidade Federal do Maranhão (UFMA), Departamento de Saúde Coletiva, São Luís (MA), Brasil.; 6 Duke Global Health Institute Duke Global Health Institute Duke University Durham(NC) Estados Unidos Duke Global Health Institute, Duke University, Durham (NC), Estados Unidos.

**Keywords:** Atenção primária à, saúde, atenção à, saúde, diagnóstico de situação de saúde, sistemas de informação geográfica, análise espacial, Primary health care, health care, health situation assessment, geographic information systems, spatial analysis, Atención primaria de salud, atención de salud, evaluación de la situación de salud, sistemas de información geográfica, análisis espacial

## Abstract

**Objetivo.:**

Apresentar metodologia para avaliação empírica da atenção primária à saúde (APS) por meio da construção de representações digitais das áreas de cobertura potencial das equipes da APS.

**Métodos.:**

Estudo de natureza metodológica. As áreas potenciais foram construídas por análise combinatória entre setores censitários e localização das unidades básicas de saúde (UBS) que apresentavam equipes de APS no Brasil. Foram utilizadas seis regras para parametrizar o algoritmo de construção das áreas potenciais. Assim, foram estipuladas seis restrições que viabilizaram o modelo utilizado: seleção de setores censitários próximos à UBS; setores contíguos; setores mutuamente excludentes; setores localizados no mesmo município da UBS; somatório de 4 500 usuários por equipe de saúde em cada UBS; e volume de população adscrita proporcional ao número de equipes de APS alocadas na UBS. A partir de 316 574 setores censitários e 39 758 UBS, foi desenvolvida uma matriz de vizinhança sobre a qual iterou um algoritmo de grafo que testava combinações de setores que atendessem simultaneamente as regras estipuladas.

**Resultados.:**

Foram definidos 1 901 114 arcos ligando 30 351 setores censitários, permitindo a construção de 26 907 áreas potenciais. A partir desse resultado é possível fazer análises inframunicipais no que tange ao monitoramento de indicadores da APS. Os parâmetros customizáveis do algoritmo podem ser ajustados para contemplar diferentes conjuntos de regras e adaptados para diferentes países.

**Conclusões.:**

O uso de abordagens amparadas em geoprocessamento pode criar condições para avaliação do impacto da APS, levando-se em conta bases de dados secundárias e com nível de análise inframunicipal, de UBS e até mesmo de equipes.

## Abstract (en)

**Objective.:**

To present a methodology for the empirical evaluation of primary health care (PHC) through the construction of digital representations of potential PHC coverage areas.

**Methods.:**

In this methodological study, potential areas were constructed by combinatorial analysis between census tracts and the location of basic health units with working PHC teams in Brazil. Six rules were used to parameterize the algorithm for the construction of potential areas. Thus, six restrictions were applied to enable the model: the selection of census tracts near the basic health unit; contiguous sectors; mutually exclusive sectors; sectors located in the same municipality of basic health units; sum of 4 500 users per health team in each unit; and volume of population ascribed proportional to the number of PHC teams allocated to the unit. Based on 316 594 census tracts and 39 758 basic health units, a neighborhood matrix was developed. To that matrix, a graph algorithm was applied to test combinations of sectors that simultaneously met the stipulated rules.

**Results.:**

A total of 1 901 114 arcs were defined, connecting 30 351 census tracts, allowing the construction of 26 907 potential areas. Based on these results, intra-municipal analyses can be performed to monitor PHC indicators. Customizable algorithm parameters can be adjusted to accommodate different sets of rules which may be adapted to different countries.

**Conclusions.:**

The use of geoprocessing approaches creates conditions for the assessment of PHC impact, based on secondary databases at various levels, such as intra-municipal, basic health unit, and even at the team level.

## RESUMEN

**Objetivo.:**

Presentar una metodología para la evaluación empírica de la atención primaria de salud (APS) a través de la construcción de representaciones digitales de las áreas de cobertura potencial de los equipos de APS.

**Métodos.:**

Estudio de tipo metodológico. Las áreas potenciales se construyeron mediante un análisis combinatorio entre los sectores censales y la localización de las unidades básicas de salud con equipos de APS que trabajan en Brasil. Se utilizaron seis reglas para parametrizar el algoritmo de construcción de las áreas potenciales. Así, se estipularon seis restricciones que viabilizaron el modelo utilizado: selección de sectores censales cercanos a la unidad básica de salud; sectores contiguos; sectores mutuamente excluyentes; sectores ubicados en el mismo municipio de la unidad básica de salud; suma de 4 500 usuarios por equipo de salud en cada unidad básica de salud; y volumen de población adscrita proporcional al número de equipos de APS asignados en la unidad básica de salud. A partir de 316 574 sectores censales y 39 758 unidades básicas de salud se desarrolló una matriz de vecindad sobre la cual se aplicó un algoritmo gráfico que evaluaba las combinaciones de sectores que cumplían simultáneamente las reglas estipuladas.

**Resultados.:**

Se definieron en total 1 901 114 arcos, que conectaron 30 351 sectores censales, lo que permitió la construcción de 26 907 áreas potenciales. Sobre la base de estos resultados, se pueden realizar análisis intramunicipales para monitorear los indicadores de APS. Los parámetros modificables del algoritmo se pueden ajustar para adaptarse a diferentes conjuntos de reglas y a diferentes países.

**Conclusiones.:**

El uso de enfoques basados en geoprocesamiento puede crear condiciones para la evaluación del impacto de la APS conforme a bases de datos secundarias y a nivel intramunicipal, de la unidad básica de salud e incluso a nivel de equipo.

Em 2014, a Organização Pan-Americana da Saúde (OPAS) conclamou os estados membros a envidarem esforços para alcançar o acesso e a cobertura universal de saúde (1). Para a consecução dos objetivos do chamado, seria fundamental promover ações intersetoriais que abordassem os desafios dos determinantes sociais em saúde, incrementassem a proteção social amparada no acesso à saúde, respeitassem as idiossincrasias culturais de cada país, reduzissem os riscos e carga de doenças, garantissem acessibilidade física e financeira a serviços de saúde e fortalecessem ações de saúde de qualidade, efetivas e oportunamente ofertadas, em termos de tempo e das especificidades locais (2, 3).

No caso da atenção primária à saúde (APS), o ajuste a especificidades locais exige um profundo conhecimento do território de atuação. A delimitação de um espaço geográfico de responsabilidade de uma determinada equipe de saúde é um dos pressupostos da APS no Brasil. Essa delimitação permite que as equipes atuem de forma a modelar a oferta de serviços de saúde de acordo com as necessidades definidas por esse espaço geográfico (4). Esse território representa, portanto, mais que uma extensão geométrica; é, também, um perfil demográfico, epidemiológico, administrativo, tecnológico, político, social e cultural (5).

Em contextos de APS, os sistemas de informação geográfica (SIG) permitem a integração de atividades de captura, armazenamento, manipulação, seleção e busca de informações e análise e apresentação de dados, possibilitando um melhor entendimento da ocorrência de eventos assim como a predição, análise de tendências, simulação de situações, planejamento e definição de estratégias de prevenção e promoção à saúde. Um estudo de revisão sobre as vantagens do uso de soluções de SIG na APS mapeou as contribuições na área (6). Esse estudo categorizou os trabalhos produzidos em três grupos: foco no desenho racional e planejamento em saúde, avaliações de acesso e cobertura da APS e, por último, esforços para compreender os padrões de utilização de serviços de saúde. Apesar do destaque à capacidade de modelagem de informações sobre a localização ótima de centros de saúde e definição de áreas de atuação e mapeamento dos elementos existentes no entorno das unidades básicas de saúde (UBS), tópicos relevantes não foram explorados, com destaque para a análise da influência de determinantes sociais em saúde e avaliações de acesso (6).

As avaliações de acesso na APS usualmente se baseiam em duas dimensões: acessibilidade e disponibilidade. A acessibilidade remete à noção de tempo de deslocamento até os serviços, ao passo que a disponibilidade se refere ao número de locais prestadores de cuidado (6). Essas duas dimensões devem consideradas em conjunto para caracterizar o que é chamado de acessibilidade espacial (7).

Atualmente, a principal técnica utilizada para mensuração de acessibilidade espacial é a *two-step floating catchment area* (2SFCA) (8). O termo *catchment area*, ou área de captura, representa uma região geográfica adjacente a um prestador de serviços, incluindo a clientela que acessa os serviços ali ofertados (9). Na área da saúde, a técnica 2SFCA foi adaptada com o intuito de permitir a criação de índices de acesso a serviços. Através de sua aplicação, é possível identificar áreas desassistidas, levando-se em consideração o volume de população existente na localidade (10).

Apesar de sua potencialidade, essa técnica apresenta limitações. A 2SFCA é pouco sensível e ignora a capacidade de prestação de cuidado nos serviços de saúde analisados. Além disso, apresenta problemas ao lidar, simultaneamente, com áreas densamente povoadas e regiões com baixa densidade populacional (10). Buscando resolver essas questões, foram desenvolvidas abordagens mais sofisticadas, como a *enhanced two-step floating catchment area* (E2SFCA), que utiliza funções de decaimento de distância ou padrões de captação variáveis para estimar os índices de acessibilidade (11). Apesar da melhoria propiciada pela utilização de funções de decaimento, o desafio de manejar satisfatoriamente áreas urbanas e rurais sem produzir distorções ainda persiste (12, 13). Sem que essas medidas sejam suficientemente precisas, as análises de acessibilidade podem gerar erros relacionados aos efeitos dos elementos geográficos sobre a saúde (14).

Considerando a relevância das técnicas de geoprocessamento para a saúde, a carência de estudos empíricos voltados para a APS utilizando SIG e os desafios para mensuração do acesso aos serviços, o presente trabalho apresenta uma nova metodologia que favorece a avaliação empírica da APS, por meio da projeção de territórios de atuação potencial de equipes de APS. A metodologia aqui proposta busca superar as limitações das técnicas atuais ao estimar as regiões de abrangência das equipes de APS utilizando parâmetros customizáveis de modo idiossincrático para a atenção primária.

## MATERIAIS E MÉTODOS

O presente estudo pode ser caracterizado como de natureza metodológica, uma vez que buscou desenvolver novas abordagens para o manejo e criação de *catchment areas* para estabelecimentos de saúde. A metodologia para a criação dessas áreas de abrangência potencial almejou desenvolver, a partir de um conjunto de parâmetros, uma representação digital que refletisse, da melhor maneira possível, as regiões adscritas às UBS prestadoras de serviços de APS no Brasil.

### Fontes de dados

Três conjuntos de dados foram utilizados: malha digital de setores censitários, volume de população vinculada a cada setor e geolocalização das UBS existentes no Brasil, considerando-se a distribuição de unidades do segundo semestre de 2015. A malha de setores censitários e o volume de população foram obtidos junto ao Instituto Brasileiro de Geografia e Estatística (IBGE) (15, 16). Os dados sobre as coordenadas de localização das UBS foram coletados junto ao Cadastro Nacional de Estabelecimentos de Saúde (CNES), disponibilizado pelo Departamento de Informática do SUS (DATASUS) (17).

### Análise de dados

As seis regras utilizadas para a parametrização do algoritmo de construção das áreas de captura foram baseadas na Política Nacional de Atenção Básica (PNAB) (18): as áreas deveriam aglutinar setores localizados o mais próximo possível da UBS em questão; o teto de população por equipe de saúde alocada em cada UBS seria de 4 500 pessoas; os setores censitários escolhidos para compor uma área deveriam ser contíguos; a escolha de setores deveria necessariamente ser feita de forma mutuamente excludente; somente setores dentro do mesmo município de localização da UBS poderiam ser testados para fins de definição da área de cobertura potencial; e o volume da população adscrita seria proporcional ao número de equipes de atenção primária alocadas na UBS.

As áreas potenciais foram delimitadas por um processo de análise combinatória entre os setores censitários adjacentes às UBS consideradas. A opção por utilizar os setores censitários se deu em decorrência da sobreposição dos mesmos com as áreas de atuação das equipes de APS. No caso brasileiro, sabemos que essa sobreposição não é perfeita, mas, muitas vezes, os setores servem como base para definição das áreas de cobertura das equipes de saúde. Além disso, para os diferentes países das Américas, há a disponibilização de setores censitários para fins de contagem populacional. Assim, a metodologia aqui desenvolvida seria replicável em outros países, desde que estejam disponíveis as coordenadas de geolocalização dos estabelecimentos de saúde, bem como uma malha digital de representação de setores censitários, densidade populacional e número de equipes de APS.

A primeira restrição se deu em função da recomendação da PNAB de que a oferta de cuidado primário seja feita de forma mais próxima possível da residência dos usuários. A segunda restrição objetivou definir como alvo de população para uma área de cobertura potencial o volume de população recomendado pelo Ministério da Saúde do Brasil. A PNAB recomenda que cada área de abrangência tenha em média 3 450 pessoas. A definição, no algoritmo, de um teto de 3 450 teria como consequência áreas com um somatório de população sempre inferior a 3 450. É impossível que todas as combinações geradas atendessem a todas as restrições e sempre obtivessem um parâmetro de população adscrita perfeitamente igual a 3 450. Assim, a solução encontrada foi estipular um volume um pouco mais alto para o algoritmo (4 500 pessoas por equipe), de forma que o volume médio de população adscrita girasse em torno do número preconizado na PNAB. A terceira restrição buscou assegurar que as áreas criadas fossem blocos compactos e não uma malha dispersa de setores censitários, potencialmente distantes da UBS. A quarta restrição se deu com o intuito de permitir que os setores fizessem parte de uma única área de abrangência. Dessa forma, não haveria o compartilhamento de áreas, o que não é preconizado pela PNAB. A quinta restrição foi estipulada para atender a lógica de organização dos serviços da APS. Como a APS é municipalizada, o impedimento de constituição de áreas em múltiplos municípios se alinha ao que foi definido pela PNAB. Por último, a sexta restrição foi parametrizada para criar áreas potenciais que levassem em conta o volume de equipes de atenção básica alocadas em cada UBS que contivesse mais de uma equipe. Assim, essas áreas teriam um volume de população maior, em função da maior capacidade de ofertar cuidados primários.

Essas restrições foram estipuladas considerando as especificidades locais da política de atenção primária brasileira. Sendo assim, nada impede que elas sejam adaptadas para atender a diferentes regras e especificidades de políticas em outros países, bem como outros níveis de atenção. A criação de áreas de captura com base em critérios complexos e não apenas em parâmetros de distância e carga populacional aglutina os esforços para superação das limitações metodológicas que existiam até então.

Em 2015, considerando um total de 39 758 UBS, foram analisados dados de 76% (30 351 UBS) que apresentaram coordenadas geográficas com grau de precisão suficiente para permitir a geolocalização. Partindo das coordenadas de localização das UBS, foi realizada uma análise de sobreposição espacial, que objetivou identificar em que setor censitário estavam localizadas as UBS selecionadas para fins de análises (19), conforme informações do IBGE (16). Levando-se em conta o setor censitário de localização de cada UBS, foi desenvolvida uma matriz de vizinhança considerando todos os 316 574 setores censitários da malha divulgada do censo de 2010 (16). Essa matriz registrou quais setores eram vizinhos, incluindo aqueles que continham UBS. Essa matriz de vizinhança foi a base para o desenvolvimento do algoritmo que percorria a malha de setores censitários, testando combinações que atendessem às seis restrições definidas. A partir dessa estrutura de relacionamento, foi estruturado um algoritmo baseado na teoria de grafos.

**FIGURA 1:**
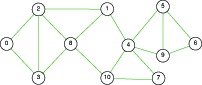
Estrutura de um algoritmo de grafo^a^

^a^ Os vértices contendo números representariam, para o presente modelo, os setores censitários; os arcos que ligam os vértices representam a estrutura de vizinhança auferida a partir da matriz. Considere-se o setor 2 como um setor contendo uma unidade básica de saúde. A lista de vizinhos de primeira ordem compreende os setores 8, 3 e 0. Caso nenhuma combinação entre esses três setores atenda as restrições do modelo, o algoritmo examina as combinações entre vizinhos de primeira e segunda ordem.

Um grafo define uma estrutura formada por dois conjuntos: um primeiro conjunto de elementos chamados de vértices e um outro conjunto denominado arcos. Cada arco associa dois vértices, sendo o primeiro a ponta de início do arco e o segundo, a ponta final (20). Um exemplo de estrutura de grafo está representado na figura 1. Os vértices, no caso da análise realizada nesse trabalho, são os setores censitários e os arcos foram definidos pela estrutura de vizinhança auferida a partir da matriz. A aplicação da abordagem de grafos para a análise de parâmetros espaciais tem recebido atenção em uma série de áreas (21). Assim, a estrutura de grafo utilizada para criação de cada área de cobertura potencial partiu do setor de localização da UBS e varreu os setores adjacentes, testando combinações que atendessem as restrições impostas ao modelo.

O algoritmo de grafos iterava sobre a matriz contendo o padrão de relacionamento de cada setor censitário de localização de uma UBS. Para cada UBS, ele testava, dentre os setores ligados diretamente por arcos ao setor de origem da UBS, combinações que atendessem, simultaneamente, todas as restrições do modelo. O algoritmo testava primeiro uma combinação de setores de vizinhos de primeira ordem. Um vizinho de primeira ordem é aquele diretamente ligado, por um arco, ao vértice de pertencimento do setor da UBS. Considerando, hipoteticamente, na figura 1, o setor 2 como um setor contendo uma UBS, a lista de vizinhos de primeira ordem compreende os setores 8, 3 e 0. Se qualquer combinação possível entre esses três setores não atendesse as restrições, então o algoritmo passava a examinar combinações entre vizinhos de primeira e segunda ordem, buscando uma combinação que atendesse às restrições. Caso todas as combinações possíveis de primeira e segunda ordem não gerassem um resultado viável, então o algoritmo passava a examinar combinações envolvendo vizinhos de ordem superior, até que as restrições fossem simultaneamente atendidas. Esse procedimento foi repetido para cada UBS no município até que todas as unidades obtivessem uma combinação plausível ou não restassem mais setores censitários disponíveis na cidade que pudessem ser testados.

## RESULTADOS

**FIGURA 2:**
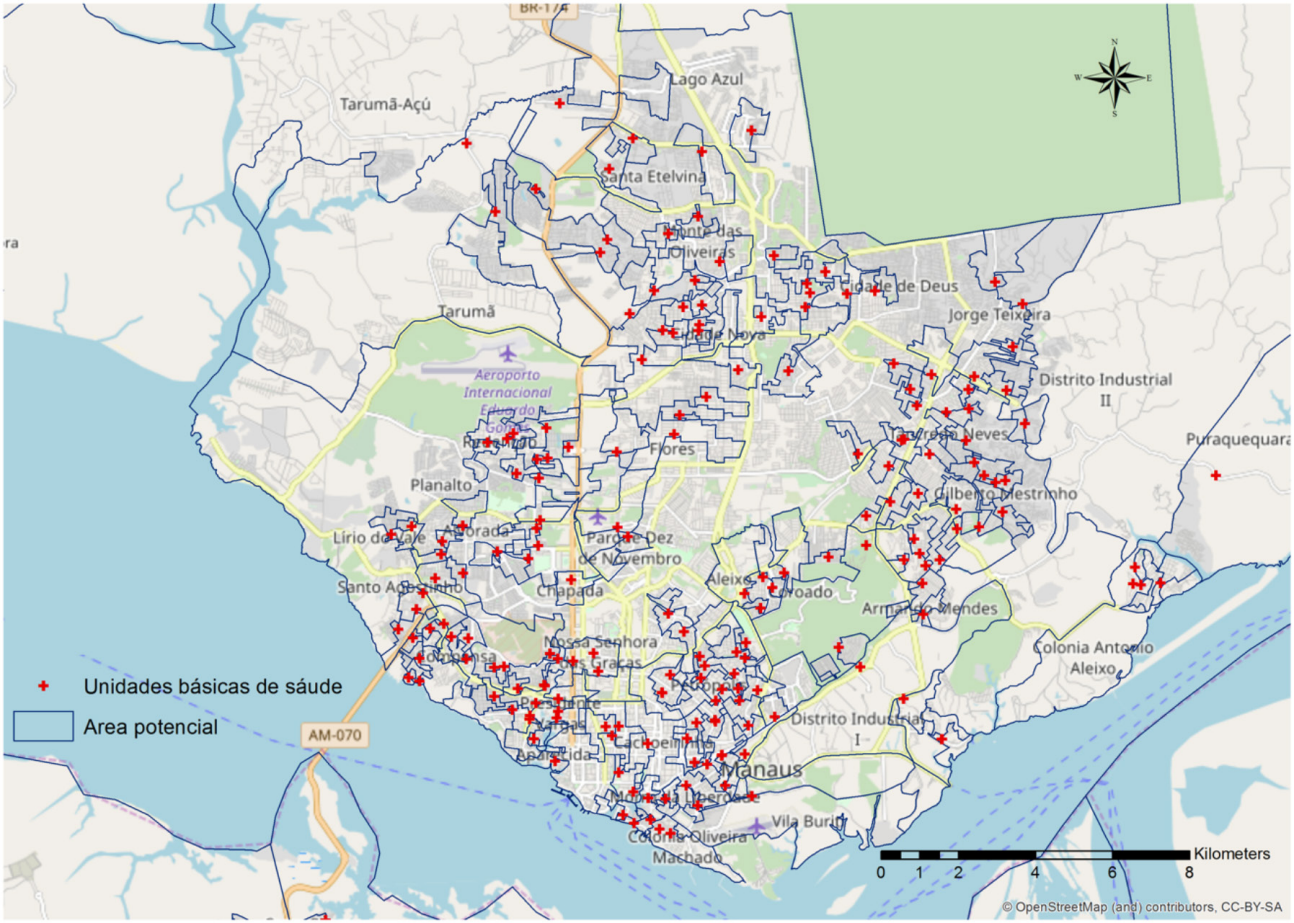
Áreas de cobertura potencial pelas equipes de atenção primária que atendiam as regras mapeadas pelo modelo para a cidade de Manaus, estado do Amazonas, Brasil, 2015

**FIGURA 3:**
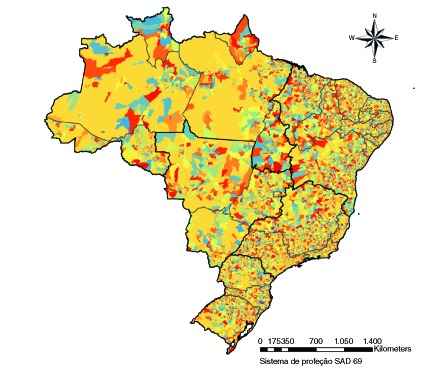
Áreas de cobertura potencial pelas equipes de atenção primária que atendiam as regras mapeadas pelo modelo, Brasil, 2015^a^

^a^ As áreas de cobertura potencial são representadas pelas manchas coloridas sobre o fundo amarelo.

Ao todo foram definidos 1 901 114 arcos, ligando 30 351 setores censitários que continham UBS. Ao final do processo de análise, foi possível obter 26 907 áreas potenciais que atenderam, simultaneamente, todos os requisitos impostos pelas restrições do modelo. A diferença entre o número de áreas potenciais (26 907) e o total de setores censitários (30 351) é reflexo da impossibilidade de atender todas as restrições para todos os pontos de UBS alocados em cada município. Uma vez esgotados os setores censitários para o teste de combinações, não era mais possível alocar UBS naquele município. A figura 2 ilustra a distribuição das áreas potencias para a cidade de Manaus, no estado do Amazonas. A figura 3 destaca as 26 907 áreas potenciais desenvolvidas para o Brasil como um todo. O volume de população por área de cobertura potencial para o Brasil foi em média 4 372 pessoas.

A tabela 1 mostra a média de população adscrita para cada área potencial. Áreas com número de pessoas acima do estipulado no modelo, de 4 500 pessoas por equipe, refletem um número maior de equipes por UBS para o estado analisado.

**TABELA 1:** Valor bruto médio de população adscrita à área de cobertura potencial por equipes de atenção primária, Brasil, 2015

Região/estado	Média de população por área	
	Média	Desvio padrão
Norte	4 856,49	5 347,64
Rondônia	6 115,03	5 308,34
Acre	3 216,54	2 072,99
Amazonas	4 663,36	3 364,93
Roraima	5 661,50	8 258,20
Pará	4 821,92	5 185,26
Amapá	6 808,20	7 823,73
Tocantins	4 186,19	6 996,78
Nordeste	3706,03	4056,00
Maranhão	4 232,69	4 005,30
Piauí	2 774,61	3 402,47
Ceará	4 169,55	4 925,48
Rio Grande do Norte	3 791,90	4 158,56
Paraíba	3 367,86	2 817,95
Pernambuco	3 999,30	3 771,52
Alagoas	3 568,85	3 991,57
Sergipe	5 373,90	6 008,61
Bahia	3 484,66	4 073,24
Sudeste	5 045,54	5 824,33
Minas Gerais	4 195,86	4 707,16
Espírito Santo	5 171,40	4 773,80
Rio de Janeiro	5 131,56	5 938,06
São Paulo	6 939,39	7 595,34
Sul	4 624,71	4 698,47
Paraná	5 431,38	5 813,85
Santa Catarina	4 238,50	4 250,57
Rio Grande do Sul	4 230,21	3 676,13
Centro-oeste	4 609,08	5 887,11
Mato Grosso do Sul	4 425,70	3 771,73
Mato Grosso	4 045,27	3 323,56
Goiás	4 625,37	6 717,03
Distrito Federal	9 813,75	12 162,43
Brasil	4 372,66	4 984,67

## DISCUSSÃO

Para que as informações de um território possam ser utilizadas para fins de planejamento de ações, é fundamental que as mesmas sejam representadas em meio digital; a análise espacial de informações exige a utilização de técnicas de geoprocessamento (22). Entretanto, muitas vezes, as representações do território e das estruturas alocadas nele são feitas de forma artesanal por agentes de saúde (23, 24). Além disso, há pouco conhecimento técnico para o manejo desse tipo de informação por parte dos profissionais de saúde (24-26). Sabe-se, contudo, que a falta de georreferenciamento de informações sobre eventos de saúde impossibilita as análises de ocorrência espacial de agravos e inviabiliza ações de vigilância epidemiológica, de mapeamento de epidemias, de prospecção de potenciais doentes sem acompanhamento e de interrelação entre aspectos do ambiente físico e determinantes sociais em saúde, entre outros (27).

A literatura evidencia que as análises de acesso por meio de técnicas de geoprocessamento são capazes de instrumentalizar ações de gestão e planejamento da oferta de cuidado (6). Apesar disso, pesam desafios quanto à criação de representações digitais para pequenas áreas, como exigem os estudos com foco na APS. As técnicas até agora disseminadas (2SFCA e E2SFCA) não são capazes de manejar, satisfatoriamente, as disparidades que podem ser observadas considerando todo o território de um país.

Nesse sentido, o presente trabalho buscou oferecer uma nova abordagem para a estimação de áreas de captura dos estabelecimentos de saúde, capaz de superar as limitações vigentes, permitindo a realização de estudos mais granulares voltados para APS. A principal contribuição do estudo se materializou através do desenvolvimento de um novo método, baseado em parâmetros multicritério. Ao propiciar a criação de áreas de cobertura potencial a partir de restrições múltiplas, o método aqui proposto supera alguns dos problemas decorrentes do uso de técnicas como a 2SFCA ou a E2SFCA. Esses dois conjuntos de técnicas são marcados pela impossibilidade de manejar desafios que extrapolem a lógica da distância e da ponderação de capacidade de oferta de serviços por população. O método desenvolvido para o presente trabalho leva em conta não só parâmetros de distância, mas também o dimensionamento de capacidade de atendimento populacional, regras de associação espacial para o meio urbano e rural e lógica regional de oferta de cuidado. Adicionalmente, abre a possibilidade para que outros elementos além desses possam fazer parte de técnicas para a criação de áreas de cobertura potencial por serviços de saúde.

Sabidamente, o acesso a serviços de saúde é balizado por outros elementos que não somente a distância e métricas de capacidade de atendimento. A necessidade de incorporar restrições mais complexas para delimitar uma área de influência de serviços de saúde é premente, considerando-se a necessidade de melhorar as estratégias para avaliação do acesso a serviços. O método aqui detalhado pode ser utilizado em outros países, com outros tipos de estabelecimentos e qualquer tipo de restrição que se mostre necessária. A definição de áreas de abrangência de hospitais pode ser feita considerando a prevalência esperada de uma doença específica em uma população e, simultaneamente, aspectos de capacidade de atendimento, como aparelhamento, número de leitos, volume de profissionais ou outras medidas de interesse, além da distância da população em relação ao estabelecimento de saúde. A parametrização de restrições complexas a serem testadas pelo algoritmo de grafos comporta qualquer conjunto de informações que esteja disponível e que permita a realização de análises combinatórias.

Para a APS, os avanços demonstrados por essa técnica criam condições para a realização de experimentos naturais, uma vez que a técnica permite a criação de linhas de base de carga de doenças, mortalidade e outros aspectos epidemiológicos de interesse público (28). A utilização de outras abordagens baseadas em geoprocessamento permite a estimação, por exemplo, de cargas de internações por condições sensíveis à APS por UBS (29). A utilização desse tipo de abordagem segundo uma lógica de série temporal cria condições para a realização de estudos quase experimentais capazes de avaliar políticas de saúde em diferentes esferas. Adicionalmente, esse tipo de abordagem permite a sobreposição de informações à área de atuação de serviços de saúde e fornece medidas mais granulares dos determinantes sociais de saúde.

A ideia de utilizar parâmetros customizáveis para direcionar a testagem de combinações entre os diferentes setores censitários permite que a abordagem definida aqui seja adaptada para outros contextos, países ou serviços de saúde. A possibilidade de definir regras específicas para um determinado contexto irá fazer com que o algoritmo de grafos mude a lógica de testagem de combinações, mas ainda assim permitindo a geração de representações digitais. Além disso, essa abordagem permite analisar dados inframunicipais, das UBS ou até mesmo de equipes de APS. Isso cria condições para melhor delinear estudos avaliativos considerando um espaço heterogêneo que pode ser encontrado em uma cidade, por exemplo. Regiões com baixa renda podem ser analisadas de modo separado de regiões com melhor padrão de rendimentos, permitindo análises mais granulares de desempenho da APS, acesso e equidade.

Apesar dos avanços explicitados, a presente abordagem apresenta limitações. A definição de áreas de pequena influência para serviços de saúde está sujeita a problemas de flutuação aleatória de pequenas áreas. A melhor abordagem para a definição de acesso a serviços perpassa a utilização de dados de prontuário eletrônico, com o registro de cada interação entre o paciente e a rede de atendimento, uma vez que esse tipo de sistema permite inferir os padrões reais de utilização de serviços e de deslocamento populacional (12). Cabe destacar ainda a caducidade dos dados referentes aos setores censitários que, em função de sua data de coleta, podem apresentar variações do ponto de vista de contingente populacional residente em cada setor. Essa diferença não invalida as análises realizadas, mas pode fazer com que os resultados auferidos com uma população atualizada sejam ligeiramente diferentes.

Em resumo, os resultados obtidos com o presente trabalho detalham uma nova abordagem metodológica para definição de áreas de captura com base em parâmetros multicritério capazes de superar algumas das limitações das técnicas até então utilizadas. O método descrito pode ser adaptado para outros tipos de serviços que não só a APS. Além disso, os passos definidos ao longo do estudo podem ser customizados para quaisquer países das Américas que possuam dados sobre parâmetros sociodemográficos e assistenciais de sua respectiva rede prestadora de cuidados.

## Contribuição dos autores.

TAHR, DGA, NCS e PVMA elaboraram as bases de dados, analisaram os dados e redigiram o manuscrito. TAHR e DGA programaram os códigos computacionais. JRNV, EBAFT e RCSQ analisaram os dados e redigiram o manuscrito. ACQB realizou a revisão do texto, que foi aprovado por todos os autores.

## Financiamento.

O autor TAHR agradece à CAPES pela bolsa de doutorado, no âmbito do qual se desenvolveu esse trabalho.

## Declaração.

As opiniões expressas no manuscrito são de responsabilidade exclusiva dos autores e não refletem necessariamente a opinião ou política da RPSP/PAJPH ou da Organização Pan-Americana da Saúde (OPAS).
